# Evaluating student perceptions of image-based audience response questions in medical education

**DOI:** 10.3389/fphar.2026.1960739

**Published:** 2026-10-13

**Authors:** Yashar Pourmoghadam, Welly Mente, Lindsay Hewitt, Kaila Polizzi, Emily H. Tran, Kenneth Thai, Hannah Chang, Tianyu Luo, John K. Cusick, Valerie A. Gerriets

**Affiliations:** 1 Department of Basic Science, California Northstate University College of Medicine, Elk Grove, CA, United States; 2 Department of Clinical and Administrative Sciences, California Northstate University College of Pharmacy, Elk Grove, CA, United States; 3 Department of Medical Education, University of California Merced, Merced, CA, United States

**Keywords:** active learning strategies, audience response system, Hotspot, medical education, pharmacology education

## Abstract

Audience response systems have been extensively utilized in medical and pharmacy education to improve learner engagement and knowledge retention. Unlike traditional ARSs that utilize text-based multiple-choice questions Hotspot is an image-based ARS integrated into PointSolutions that allows students to answer questions by selecting areas of interest on images and figures, rather than choosing from multiple-choice options. The purpose of this study was to evaluate student perceptions of integrating Hotspot into pharmacology and biomedical curricula, including perceived engagement, learning reinforcement, usability and preferences relative to traditional MCQs. Faculty at California Northstate University incorporated Hotspot questions into the lectures of first- and second-year courses across three matriculating classes. Following these sessions, students completed an anonymous survey. Survey items assessed student perceptions of engagement, perceived learning, and ease of use on a seven-point Likert scale. Learners reported that Hotspot technology made it easier to pay attention during lectures (median Likert score of six out of seven [interquartile range (IQR) 5–7]; n = 99). They felt the questions were a good use of class time (6 out of 7 [interquartile range 6–7]; n = 99) and indicated that the interactive format challenged them and reinforced their understanding better than MCQs (6 out of 7 [IQR 5–7]; n = 99). Overall, learners expressed a preference for Hotspot over traditional MCQs and reported that this approach was more engaging. These findings support the feasibility and learner acceptability of incorporating image-based active learning strategies and provide evidence to support the adoption of innovative instructional approaches in medical and pharmacology education.

## 1 Introduction

Traditionally, medical and pharmacy undergraduate education has utilized didactic lecturing as the primary format for learning basic biomedical science topics. More recently, there has been a shift from lecturing to more active and engaging styles of teaching. One such form of active learning, audience response systems (ARS), are now widely used in medical and pharmacy education. An ARS is an interactive technology that allows the instructor to engage with the audience through asking questions, typically displayed on a screen visible by the entire group of learners. The learners can then provide a response using clickers or their own personal device (laptop, cell phone or tablet) and the results are collected, combined and presented to the class in a visual format, such as a histogram (Morrell and Joyce, 2015; Jandu, 2018; Kay and LeSage, 2009). The results and concepts described in the question can then be discussed by the instructor and learners to reinforce concepts and clarify any misconceptions. ARSs have been extensively studied, and systematic reviews on the topic demonstrate improvements in learner engagement, participation and knowledge outcomes (Kay and LeSage, 2009; Mains et al., 2015; Serrada-Sotil et al., 2025).

Previous studies have shown that medical and pharmacy students perceive ARSs as superior to verbal questioning, help learners pay attention and support motivation through immediate formative feedback (Iqbal et al., 2021; Cain et al., 2009; Bawazeer et al., 2023; David and Netta, 2023; Goyal et al., 2023). Research also suggests that learners report favorable perceptions of ARS use in lectures, with many studies reporting that students preferred lectures incorporating ARS over traditional didactic formats (Kay and LeSage, 2009; Cain et al., 2009; Nelson et al., 2012; Lymn and Mostyn, 2010). Other studies have reported that both students and faculty members agree that ARSs are easy to use and improve participation (Iqbal et al., 2021; Alexander et al., 2009). There are a variety of platforms that can be used for ARSs, including PointSolutions (formerly Turning Point), Kahoot!, Poll Everywhere and others (Jandu, 2018; Serrada-Sotil et al., 2025; Awan, 2022).

The vast majority of the ARSs reported in the literature utilize a text-based multiple-choice response format. This can be beneficial for students, as it is a similar format to commonly used assessment tools (Parekh and Bahadoor, 2024). However, multiple-choice questions (MCQs) can be susceptible to cueing effects, with recognition rather than understanding driving responses and many MCQs tend to utilize lower order thinking skills (Awan, 2022; Parekh and Bahadoor, 2024; Sher et al., 2026). In addition, many of the biomedical topics taught in medical and pharmacy education, including pharmacology, utilize visual and spatial representation of mechanisms of action, signaling pathways and pharmacokinetic relationships that may not be well represented as MCQs with predefined answer choices.

Hotspot is an image-based ARS modality integrated into PointSolutions that allows students to answer questions by selecting regions of interest directly on diagrams, figures, histologic images, pathways, and other visual educational content. Unlike traditional MCQ used in ARSs, Hotspot questions require students to spatially identify structures or concepts within an image-based framework. Although image-based ARSs have been developed, published evaluations have focused primarily on the technical implementation of the technology or radiology resident education, and therefore little is known about learning perceptions of image-based ARSs in undergraduate medical and pharmacy education (van Ooijen et al., 2011; Jorritsma et al., 2015). Image-based Hotspot questions represent an emerging yet unexplored extension of ARS technology (Awan, 2022). Therefore, this study aimed to evaluate medical student perceptions of image-based Hotspot questions embedded within a large-lecture format, examining student perception of engagement and perceived learning.

## 2 Methods

### 2.1 Materials and training

Image-based ARS questions were administered using the Hotspot feature in PointSolutions (Echo360, Dulles, VA, United States), allowing for real-time collection of student responses through web-enabled personal devices. Pointsolutions was available to all students through individual subscriptions funded by California Northstate University, allowing all learners to access the platform during learning sessions. The medical school faculty members involved in this study were exposed to Hotspot technology and features during a faculty development seminar by the pharmacy school faculty member involved in this study.

### 2.2 Participants

Participants consisted of three cohorts of first-year medical students enrolled at California Northstate University College of Medicine (CNUCOM) between 2023 and 2025 (Class of 2027, Class of 2028 and Class of 2029), and one cohort of first-year pharmacy students enrolled at CNU College of Pharmacy in 2025. Of the 99 CNUCOM participants, 36 identified as male, 57 as female, 1 as non-binary and five students preferred not to answer the question. Eight students were between the ages of 18–21, 62 between the ages of 22–24, 25 students were between ages 25–28, three students were between 28 and 32 and one student preferred not to answer the question. Participation in the study was voluntary and anonymous and was not associated with course grades or academic standing. To encourage participation, students who completed the survey were eligible to enter a raffle for small university prizes, including jackets, hoodies, backpacks, hats, and water bottles. Survey responses were only collected once from each participant.

### 2.3 Study design

This study received approval from the California Northstate University Institutional Review Board (IRB). This single-group observational study evaluated student perceptions following exposure to image-based Hotspot questions that were incorporated into regularly scheduled pharmacology, immunology, cell biology, and biochemistry lectures during the academic year. The study did not include a concurrent control or traditional MCQ comparator group. After participating in a lecture in which Hotspot was incorporated, students were invited to complete an anonymous electronic survey. Prior to initiation of the survey, informed consent was obtained from participating students. Two faculty members in the College of Medicine incorporated Hotspot activities within pharmacology and immunology lectures for medical students and one faculty member in the College of Pharmacy incorporated Hotspot questions into clinical and administrative sciences didactic courses for pharmacy students (Supplementary Table S1). Hotspot activities were incorporated into both in-person and hybrid learning environments. Faculty members determined the number, content and timing of Hotspot questions based on instructional needs; therefore, implementation was not standardized across sessions or courses.

Using personal electronic devices, such as laptops, tablets, and mobile phones, students were asked to log into their PointSolutions account and access the Hotspot questions. Within the platform, they were asked to identify regions of interest directly within visual educational content pertinent to the lecture they were attending (example in Figure 1A). After a response interval, determined at the discretion of the faculty member depending on question complexity, correct answers were revealed, and the aggregated anonymous response distributions were displayed. Green dots indicate that the student selected the correct area or region, whereas red dots indicate an incorrect selection (Figure 1B). Furthermore, as shown in Figure 1B, a shaded area was made visible to the students clearly indicating which answers were scored as correct. Instructors then facilitated discussion following each Hotspot question and reviewed the rationale underlying the correct responses, using the students’ responses to gauge the collective understanding of the learners and identify areas of confusion and misunderstanding. This allowed the educator to reinforce conceptual understanding of topics and provide immediate formative feedback.

**FIGURE 1:**
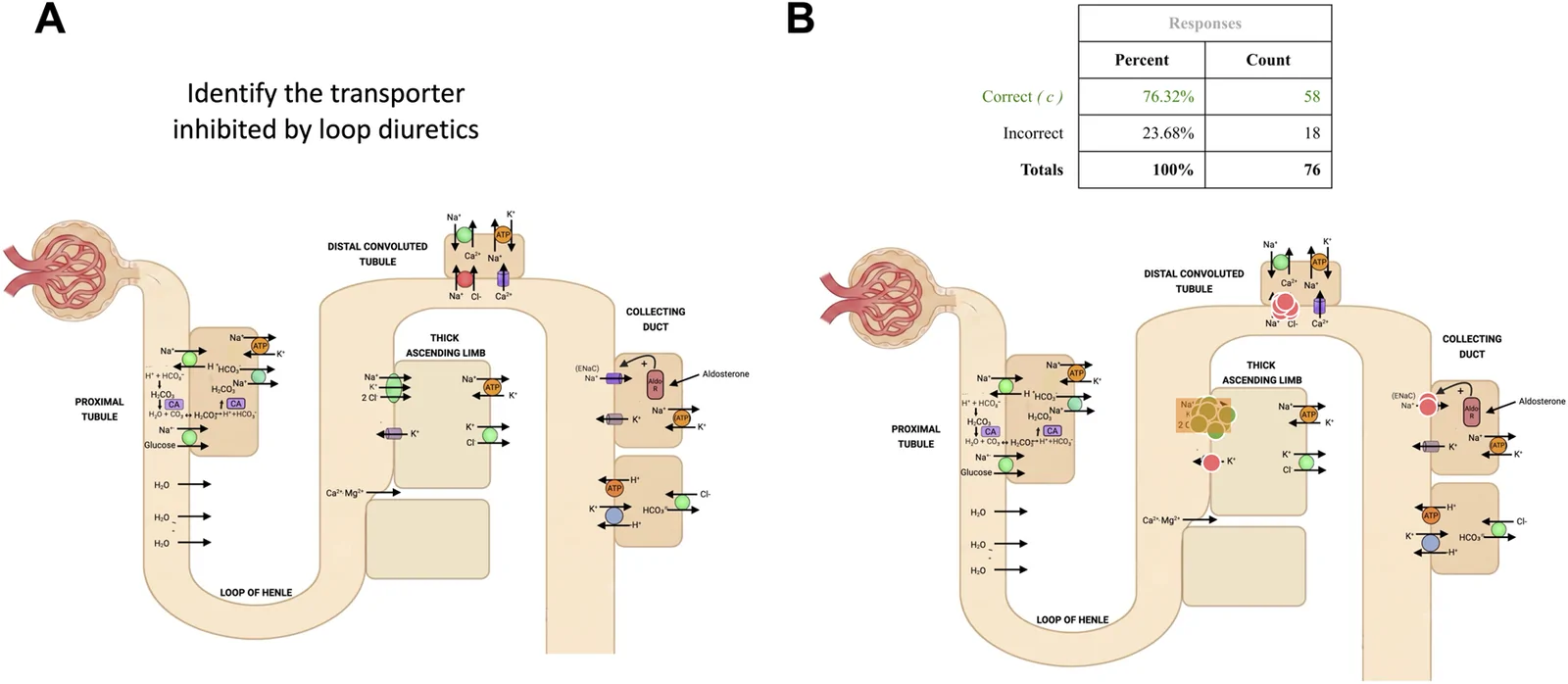
Example of a hotspot question administered through pointsolutions. **(A)** Example Hotspot question in which students were shown an image of nephron and asked to identify the transporter inhibited by loop diuretics by selecting the appropriate location directly on the image. **(B)** Example aggregated anonymous student responses displayed following question submission. Green dots represent correct responses within the predefined target region (orange box), whereas red dots represent incorrect responses. Created in BioRender. Gerriets, V. (2026), https://BioRender.com/x6uqhdh.

### 2.4 Survey administration

Following participation in lectures utilizing Hotspot, student perception was assessed through an anonymous electronic survey administered using the surveying platform SurveyMonkey. Student researchers participating in the project administered and collected survey responses from participants.

Survey questions were developed by the investigators to assess student perception of the Hotspot system regarding their engagement, attentiveness, educational value, ease of use, perceived learning enhancement, exam preparedness, and preferences for future incorporation of such activities (Supplementary Table S2). Eight survey questions were administered and assessed using a seven-point Likert scale, ranging from strongly disagree (1) to strongly agree (7). The survey questions were not piloted previously and prior exposure to ARSs was not assessed.

Student responses were collected anonymously, and no identifying information was linked to participant responses. During the duration of this multiyear study, a total of 99 survey responses were collected from medical students. Medical student participants included 17 responses from the Class of 2027 (AY2023-2024), 48 responses from the Class of 2028 (AY2024-2025), and 34 responses from the Class of 2029 (AY2025-2026) (Supplementary Table S3). Three first-year pharmacy students from the Class of 2028 (AY2025-2026) also completed the survey. Because the pharmacy student sample size was insufficient for meaningful statistical analysis, quantitative analyses were restricted to medical student participants.

### 2.5 Data analysis

Data collected from SurveyMonkey was exported into Microsoft Excel for organization and analysis. Survey responses were compiled and converted into numerical Likert-scale values for quantitative analysis of all academic years. Statistical analysis was then performed on the aggregated survey responses using the open-source Integrated Development Environment (IDE) RStudio.

Median Likert scores and IQRs (interquartile ranges) were calculated for each survey item, and student responses were then additionally stratified by academic year, age, and gender to examine consistency of student perceptions throughout the student population and duration of the study. Differences in responses across stratified groups were calculated using separate Kruskal-Wallis tests for each of the 8 Likert-scale survey items, with Benjamini-Hochberg (BH) adjustments for multiple comparisons to control the false discovery rate (FDR). For survey items that remained significant after adjustment, Dunn’s *post hoc* tests with the BH correction were performed to identify pairwise cohort differences. A p-value less than 0.05 was considered statistically significant. The primary objective of this study was to evaluate student perceptions and the feasibility of implementing Hotspot technology within medical education; henceforth, analyses focused primarily on descriptive outcomes rather than hypothesis-driven comparisons. Results are presented as median Likert-scale scores with corresponding measures of variability.

Internal consistency among the five related survey items assessing Engagement and Educational Value (“easier to pay attention”; “preferred Hotspot over MCQs”; “would like Hotspot used more frequently”; “good use of class time”; and “would attend more frequently”) was assessed using Cronbach’s alpha (Microsoft Excel).

## 3 Results

### 3.1 Student perceptions of Hotspot technology

Across all survey responses, medical students reported favorable perceptions regarding the incorporation of Hotspot technology into lecture materials (Figure 2; Supplementary Figure S1). Overall, students indicated that Hotspot was easy to set up and use on their personal electronic devices (median seven out of 7 (IQR 6–7); n = 86) and was integrated smoothly into the classroom setting, promoting active participation. Students also reported that Hotspot questions were a good use of instructional time (6 out of 7 (IQR 6–7); n = 99) and it was easier to pay attention during class sessions that incorporated Hotspot activities (6 out of 7 (IQR 6–7); n = 99) (Figure 2; Supplementary Table S4).

**FIGURE 2:**
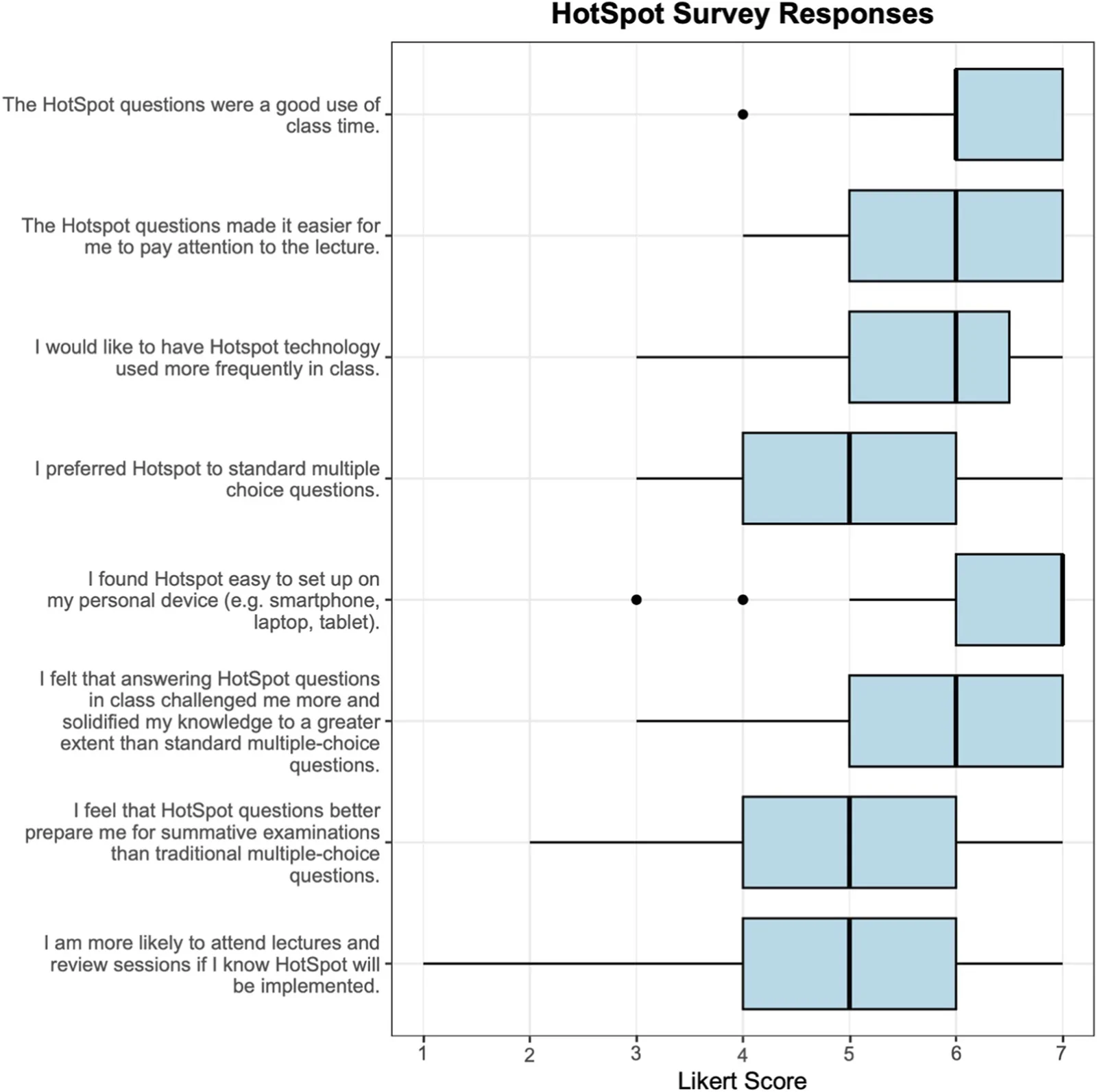
Student perceptions of Hotspot technology. First- and second-year medical students (n = 99) from three cohorts completed an anonymous survey following participation in lectures incorporating Hotspot activities. Responses were recorded on a seven-point Likert scale ranging from strongly disagree (1) to strongly agree (7). Shown are box-and-whisker plots representing student responses to survey items. Boxes represent the interquartile range (IQR), the center line indicates the median, whiskers extend to 1.5 × IQR, and points denote outliers.

Medical students indicated a preference for more frequent use of Hotspot technology in class (6 out of 7 (IQR 5–6.5); n = 99) and that they were more likely to attend lectures incorporating Hotspot activities (5 out of 7 (IQR 4–6); n = 98) (Figure 2; Supplementary Table S4). They also reported that the interactive format of Hotspot challenged them, solidifying the material to a greater extent than traditional MCQs (6 out of 7 (IQR 5–7); n = 99) and that Hotspot activities enhanced active engagement with lecture material and supported learning reinforcement, leading to students feeling more prepared for their exams (5 out of 7 (IQR 4–6); n = 99). Pharmacy students were also surveyed and the responses from the small pharmacy student subgroup were qualitatively similar to those observed among medical students (7 out of 7 [IQR 6–7] across all survey items; n = 3). Collectively, survey responses suggested that students perceived Hotspot technology as educationally beneficial, accessible, and engaging.

### 3.2 Engagement and perceived learning outcomes

Next, we examined the percentage of medical students that agreed (somewhat agreed, mostly agreed and strongly agreed) or were neutral/disagreed (neither agreed or disagreed, somewhat disagreed, mostly disagreed or strongly disagreed) with each survey item and categorized them into Engagement and Educational Value, Perceived Learning, and Other (Table 1). Of the survey items assessing Engagement and Educational Value, 99% of students agreed that the use of Hotspot questions was a good use of class time and 90.9% agreed that the use of hotspot questions made was easier to pay attention to lecture. Additionally, 83.8% agreed that they would like to use hotspot technology more frequently in class, 73.7% preferred Hotspot to traditional MCQs, and 59.2% agreed that they are more likely to attend lectures and review sessions if Hotspot will be implemented. Internal consistency of the five-item Engagement and Educational Value survey items was assessed using Cronbach’s alpha. The items demonstrated good internal consistency (Cronbach’s α = 0.84), exceeding the commonly used threshold of 0.70 for acceptable internal consistency. We also examined Perceived Learning and found that 79.8% of students agreed that the use of Hotspot questions challenged them and solidified the content more than MCQs and 64.6% agreed that Hotspot questions better prepared them for exams than traditional MCQs. Finally, 95.3% agreed that Hotspot was easy to set up on their personal device. Overall, students reported that use of an image-based ARS was easy for them to set up, engaging, and the majority of students felt that Hotspot reinforced their learning.

**TABLE 1:** Student perceptions of image-based hotspot questions by survey domain.

Category	Survey item	Strongly/mostly/somewhat disagree/neither agree or disagree	Strongly/mostly/somewhat agree
*Engagement and Educational Value*	The Hotspot questions made it easier for me to pay attention to the lecture	9.1%	90.9%
	The HotSpot questions were a good use of class time	1.0%	99.0%
	I would like to have Hotspot technology used more frequently in class	16.2%	83.8%
	I preferred Hotspot to standard multiple choice questions	26.3%	73.7%
	I am more likely to attend lectures and review sessions if I know Hotspot will be implemented	40.8%	59.2%
*Perceived Learning*	I felt that answering Hotspot questions in class challenged me more and solidified my knowledge to a greater extent than standard multiple-choice questions	20.2%	79.8%
	I feel that Hotspot questions better prepare me for summative examinations than traditional multiple-choice questions	35.4%	64.6%
*Other*	I found Hotspot easy to set up on my personal device (e.g., smartphone, laptop, tablet)	4.7%	95.3%

Responses were collected from first- and second-year medical students (n = 99) following participation in lectures incorporating Hotspot technology over a 3-year period. Survey items were grouped into three domains: Engagement and educational value, perceived learning, and other. Responses were dichotomized into agreement (somewhat agree, mostly agree, strongly agree) and non-agreement (neutral, somewhat disagree, mostly disagree, strongly disagree), and values are presented as the proportion of respondents within each category.

### 3.3 Longitudinal trends

Overall, there were no statistically significant differences identified in survey responses across the 3 years that the survey was conducted (p > 0.05) for seven of the eight items after Benjamini-Hochberg correction, suggesting that there were consistently positive student perceptions of Hotspot technology observed over the longitudinal study period (Figure 3). There was only one survey item that showed a significant difference between cohorts. Preference for Hotspot over traditional multiple-choice questions differed significantly across cohorts (H(2) – 11.0, unadjusted p = 0.004, FDR-adjusted p = 0.0329). Dunn’s *post hoc* analysis demonstrated lower preference among the Class of 2029 compared with both the classes of 2027 (adjusted p = 0.00577) and 2028 (adjusted p = 0.0233), whereas the Class of 2027 and 2028 cohorts did not differ significantly (adjusted p = 0.179). Median preference scores were 6 (IQR 5–7), 6 (IQR 5–6) and 5 (IQR 4–5.75) for the Classes of 2027, 2028 and 2029, respectively. All groups remained above the neutral midpoint of the Likert scale, suggesting an overall positive perception across cohorts.

**FIGURE 3:**
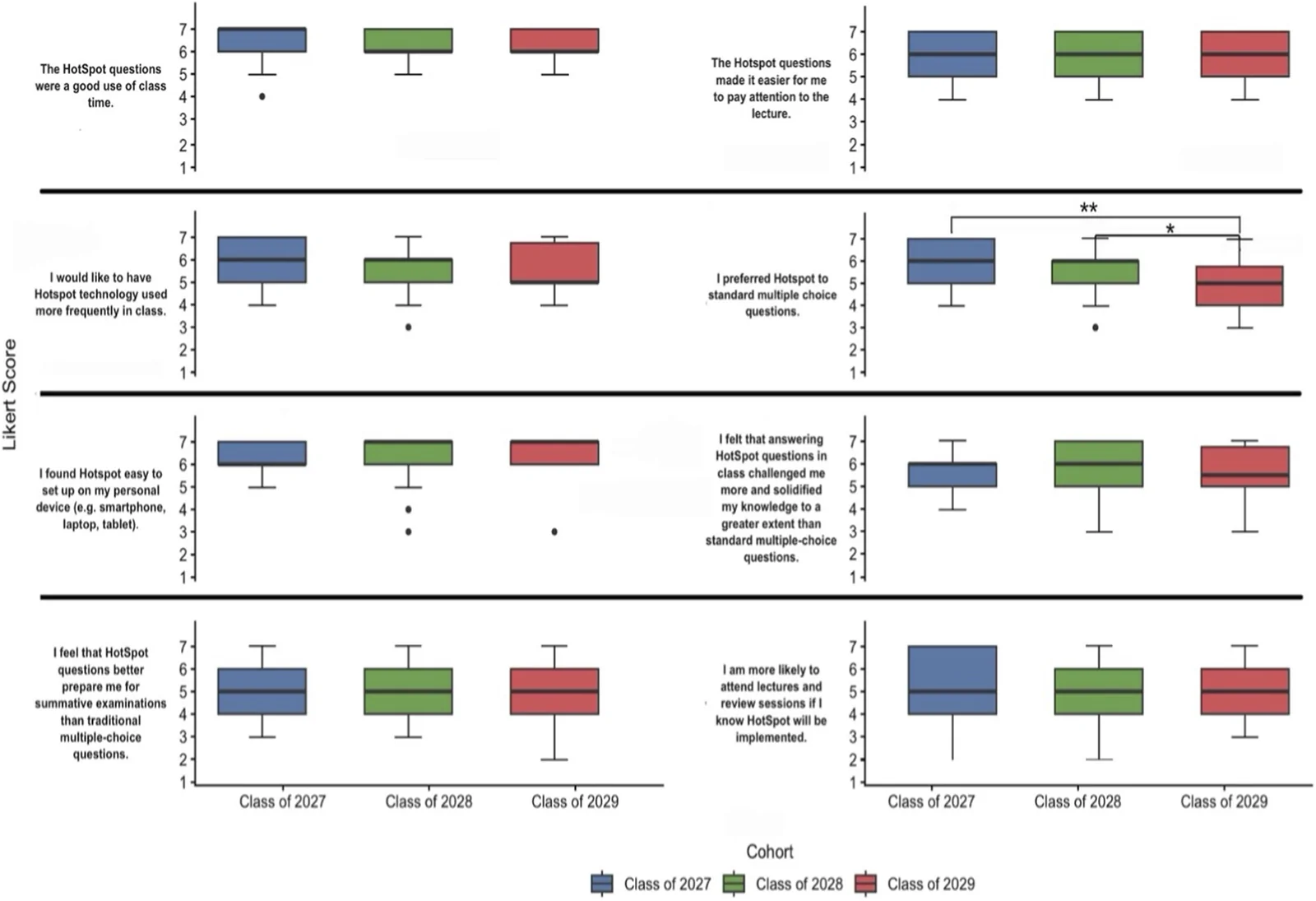
Comparison of hotspot survey responses by cohort. Medical students from the Class of 2027, Class of 2028, and Class of 2029 completed an anonymous survey using a seven-point Likert scale following participation in lectures incorporating Hotspot activities. Boxplots represent the median and interquartile range (IQR), with whiskers extending to 1.5 × IQR; individual points represent outliers. Differences between cohorts were examined using the Kruskal-Wallis test with Benjamini-Hochberg (BH) adjustments for multiple comparisons. For the survey item demonstrating a significant overall cohort difference, pairwise comparisons were performed using Dunn’s *post hoc* test with BH adjustment. Preference for hotspot over standard multiple-choice questions differed significantly by cohort, with the Class of 2029 reporting lower preference than both the Class of 2027 (adjusted p = 0.0058) and Class of 2028 (adjusted p = 0.0233). **p < 0.01; *p < 0.05.

We also examined whether there were differences in survey responses across student age and gender and did not find any statistically significant differences. Across all 3 years, students consistently rated Hotspot activities as engaging, educationally valuable, and preferable to traditional, passive instructional approaches. These findings suggest that the incorporation of an image-based interactive audience response system may present a sustainable learning strategy for pre-clinical health education learning.

## 4 Discussion

This study sought to evaluate student perceptions of an image-based audience response system (ARS), Hotspot, in undergraduate medical and pharmacology education. Across three medical school cohorts, students consistently reported that the use of Hotspot questions were engaging, easy to use and educationally valuable. These findings suggest that image-based audience response questions may be a practical active learning strategy that is well received in preclinical biomedical education.

Positive responses from students are consistent with prior literature showing that ARSs can improve engagement, participation, and perceived learning in health professions education (Serrada-Sotil et al., 2025; Cain et al., 2009; Bawazeer et al., 2023; David and Netta, 2023; Goyal et al., 2023; Nelson et al., 2012; van Ooijen et al., 2011; Miller et al., 2003). However, unlike conventional ARS platforms that primarily rely on traditional text-based multiple-choice formats, image-based ARSs require learners to identify structures, pathways and regions of interest directly within visual representations in figures and images (Awan, 2022). The visual-spatial format encourages spatial reasoning and visual interpretation rather than recognition of a correct answer from a predefined list of options. This aligns well with many concepts taught in preclinical pharmacology and more broadly in biomedical science, where signaling pathways, histological images and mechanisms of action are commonly used to enhance learning and organize knowledge. As such, image-based questioning may better mirror the cognitive processes required during medical training than conventional multiple-choice questions (MCQs).

Beyond its use as a standalone audience response modality, Hotspot technology can also serve as a flexible instructional platform upon which educators can build increasingly interactive and engaging learning experiences. Within the PointSolutions platform, Hotspot questions can be incorporated into team competitions, in which leaderboards can be utilized to track scoring of student responses either competing individually or as teams. The use of leaderboards can create a competitive, yet friendly and fun atmosphere, especially if the points in the game do not influence the grades of the students (Pettit et al., 2015; Cusick, 2016). Previous studies have indicated the ability to wager questions is very popular with students, as one study showed that 74% of students felt that wager questions were somewhat or very engaging, and another study reported that students felt the ability to wager points on a question occasionally helped keep students engaged, especially for teams at the bottom of the leaderboard (Likert Scale score of 5.7 out of 7) (Pettit et al., 2015; Chou et al., 2026). During this study, Hotspot questions were often used as wager questions, and this generated a lot of excitement and enthusiasm among the students in the classroom at the conclusion of a learning session. These questions with gamification features may further enhance motivation, participation, and classroom engagement while maintaining the formative nature of the learning activity. Although these approaches were not specifically evaluated in the present study, they illustrate the versatility of interactive image-based ARSs and provide opportunities for future educational innovation.

A key finding from the study was that students self-reported that Hotspot enhanced engagement and reinforced their learning but did not report using it as a determinant of test performance or lecture attendance. Although students reported that Hotspot helped them prepare better for exams, that item received a lower rating than items that perceived engagement. The likelihood of attending lectures was also rated comparatively lower. This distinction is notable, as it suggests that learners may value Hotspot more in the learning process itself rather than for studying and improvements in assessment outcomes. Another important finding of this investigation was the consistency of student perceptions across three distinct cohorts of learners. With the exception of one survey item comparing Hotspot with traditional MCQs, no statistically significant differences were observed between cohorts, suggesting that favorable perceptions of Hotspot were sustained across the 3-year study period. This reproducibility supports the feasibility of incorporating image-based audience response activities into medical and pharmacology curriculum as a viable educational strategy.

Despite these findings, several limitations should be considered. Objective learning outcomes, including exam performance and long-term knowledge retention were not collected as part of this study. Future studies will examine the effects of image-based ARSs on knowledge retention and exam performance compared with traditional ARSs and other instructional methods. Prior exposure to ARSs was not assessed and students may have had different exposure to ARSs previously that may have influenced their perceptions of Hotspot. Additionally, because this was a single-group perception study without a concurrent MCQ comparator group, responses to survey items comparing Hotspot with traditional MCQs reflect students’ subjective perceptions of the technology, and therefore additional studies with comparator groups would be needed to further validate the findings. This study was also conducted at a single institution, and the small number of pharmacy respondents prevented meaningful subgroup analysis, which may limit generalizability. Additionally, the survey instrument used was developed by faculty for this study and the specific questions were not validated against established engagement or learning scales. Survey participation was voluntary and the response rate varied across cohorts (Supplementary Table S3); additionally, not all participants responded to every survey item, resulting in item-specific sample sizes ranging from 86 to 99. Therefore, the findings may be subject to both cohort- and item-level nonresponse bias.

Overall, these findings provide evidence to support the adoption of innovative, active-learning instructional approaches in medical and pharmacology education. Hotspot was well received by students and represents a promising image-based extension of traditional ARS technology. Hotspot and other image-based ARSs may provide educators with an alternative strategy to promote active learning and support learner engagement within large classroom instruction.

## Data Availability

The raw data supporting the conclusions of this article will be made available by the authors, without undue reservation.
