# Supplementary material for: Evaluating student perceptions of image-based audience response questions in medical education
Source: Front Pharmacol. 2026 Oct 13;17:1960739. doi: 10.3389/fphar.2026.1960739 (PMC13710034; doi:10.3389/fphar.2026.1960739)
Supplement: Supplementary file 1 [file Table1.docx]

Supplementary Material

# 1 Supplementary Figures and Tables

## 1.1 Supplemental Figure 1

**Supplemental Figure 1. Student perceptions of Hotspot technology.** First- and second-year medical students from three cohorts completed an anonymous survey following participation in lectures incorporating Hotspot activities. Responses were recorded on a 7-point Likert scale ranging from strongly disagree (1) to strongly agree (7). Bars represent the mean Likert score for each survey item and higher scores represent greater agreement with the survey statement.

## 1.2 Supplemental Table 1

| Program | Course | Discipline | Session Titles | # Hotspot questions |
| --- | --- | --- | --- | --- |
| Medicine | Foundations of Clinical Medicine | Pharmacology | Introduction to Antibiotics | 5 |
| Medicine | Foundations of Clinical Medicine | Immunology | Antibodies and Antigens, MHC and Antigen Presentation, Immune Receptors and Lymphocyte Activation | 8 |
| Medicine | Integumentary and Musculoskeletal System | Pharmacology | Management of Infectious Diseases of the Musculoskeletal System | 8 |
| Medicine | Renal System | Pharmacology | Treatment of UTI | 8 |
| Medicine | Neuroscience | Immunology | Autoimmune Diseases of the Nervous System | 5 |
| Pharmacy* | Longitudinal Practicum II | Clinical and Administrative Sciences | Aseptic Technique/IV Sterile Compounding Procedures, Aseptic Technique/IV Sterile Compounding Regulations, Aseptic Technique/ IV Sterile Compounding Assessment Session | 6 |

**Supplemental Table 1. Implementation of Hotspot Questions Across Courses and Disciplines.** Hotspot questions were incorporated into regularly scheduled medical and pharmacy lectures. The table summarizes the course, disciplinary area, session titles and number of Hotspot questions administered during each session. The number of questions varied across sessions based on content and educational needs as determined by faculty. *Because the pharmacy student sample size was insufficient for meaningful statistical analysis, quantitative analyses were restricted to medical student participants.

## 1.3 Supplemental Table 2

What is your age?

a. 18-21yo

b. 21-24yo

c. 25-28yo

d. Greater than 28yo

e. Decline to state

What gender do you identify with?

a. Male

b. Female

c. Other

d. Decline to state

I found Hotspot easy to set up on my personal device (e.g. smartphone, laptop, tablet)

a. 1 (strongly disagree)

b. 2 (mostly disagree)

c. 3 (somewhat disagree)

d. 4 (sometimes)

e. 5 (somewhat agree)

f. 6 (mostly agree)

g. 7 (strongly agree)

h. N/A

The Hotspot questions made it easier for me to pay attention to the lecture.

a. 1 (strongly disagree)

b. 2 (mostly disagree)

c. 3 (somewhat disagree)

d. 4 (sometimes)

e. 5 (somewhat agree)

f. 6 (mostly agree)

g. 7 (strongly agree)

h. N/A

I preferred Hotspot to standard multiple-choice questions (e.g. Kahoot)

a. 1 (strongly disagree)

b. 2 (mostly disagree)

c. 3 (somewhat disagree)

d. 4 (sometimes)

e. 5 (somewhat agree)

f. 6 (mostly agree)

g. 7 (strongly agree)

h. N/A

I felt that answering Hotspot questions in class challenged me more and solidified my knowledge to a greater extent than standard multiple-choice questions.

a. 1 (strongly disagree)

b. 2 (mostly disagree)

c. 3 (somewhat disagree)

d. 4 (sometimes)

e. 5 (somewhat agree)

f. 6 (mostly agree)

g. 7 (strongly agree)

h. N/A

I would like to have Hotspot technology used more frequently in class.

a. 1 (strongly disagree)

b. 2 (mostly disagree)

c. 3 (somewhat disagree)

d. 4 (sometimes)

e. 5 (somewhat agree)

f. 6 (mostly agree)

g. 7 (strongly agree)

h. N/A

I feel that Hotspot questions better prepare me for summative examinations than traditional multiple-choice questions.

a. 1 (strongly disagree)

b. 2 (mostly disagree)

c. 3 (somewhat disagree)

d. 4 (sometimes)

e. 5 (somewhat agree)

f. 6 (mostly agree)

g. 7 (strongly agree)

h. N/A

The Hotspot questions were a good use of class time.

a. 1 (strongly disagree)

b. 2 (mostly disagree)

c. 3 (somewhat disagree)

d. 4 (sometimes)

e. 5 (somewhat agree)

f. 6 (mostly agree)

g. 7 (strongly agree)

h. N/A

I am more likely to attend lectures and review sessions if I know Hotspot will be implemented.

a. 1 (strongly disagree)

b. 2 (mostly disagree)

c. 3 (somewhat disagree)

d. 4 (sometimes)

e. 5 (somewhat agree)

f. 6 (mostly agree)

g. 7 (strongly agree)

h. N/A

**Supplemental Table 2. Survey Questions.** Participants consisted of three cohorts of first-year medical students enrolled at California Northstate University College of Medicine (CNUCOM). Participants completed a survey following participation in a lecture utilizing the image-based audience response system Hotspot consisting of demographic questions about age and gender followed by 8 questions on a 7-point Likert scale.

## 1.4 Supplemental Table 3

| **Cohort** | **Eligible** | **Participants** | **Response Rate** |
| --- | --- | --- | --- |
| Class of 2027 | 118 | 17 | 14.4% |
| Class of 2028 | 118 | 48 | 40.7% |
| Class of 2029 | 118 | 34 | 28.8% |

**Supplemental Table 3. Survey Participation and Response Rates by Cohort.** Participants consisted of three cohorts of first-year medical students enrolled at California Northstate University College of Medicine (CNUCOM) between 2023 and 2025. All of the first-year medical students were eligible to participate; study participation was voluntary and the number of participants per cohort and response rates are shown.

**1.5	Supplemental Table 4**

| **Question** | **n** | **Median** | **Q1** | **Q3** | **IQR** |
| --- | --- | --- | --- | --- | --- |
| The Hotspot questions were a good use of class time. | 99 | 6 | 6 | 7 | 1 |
| The Hotspot questions made it easier for me to pay attention to the lecture. | 99 | 6 | 5 | 7 | 2 |
| I would like to have Hotspot technology used more frequently in class. | 99 | 6 | 5 | 6.5 | 1.5 |
| I preferred Hotspot to standard multiple choice questions. | 99 | 5 | 4 | 6 | 2 |
| I found Hotspot easy to set up on my personal device (e.g. smartphone, laptop, tablet). | 86 | 7 | 6 | 7 | 1 |
| I felt that answering Hotspot questions in class challenged me more and solidified my knowledge to a greater extent than standard multiple-choice questions. | 99 | 6 | 5 | 7 | 2 |
| I feel that Hotspot questions better prepare me for summative examinations than traditional multiple-choice questions. | 99 | 5 | 4 | 6 | 2 |
| I am more likely to attend lectures and review sessions if I know Hotspot will be implemented. | 98 | 5 | 4 | 6 | 2 |

**Supplemental Table 4. Student perceptions of Hotspot technology. First- and second-year medical students from three cohorts completed an anonymous survey following participation in lectures incorporating Hotspot activities. Responses were recorded on a 7-point Likert scale ranging from strongly disagree (1) to strongly agree (7). Shown are the number of respondents of each question (n), media, first quartile (Q1; 25^th^ percentile), third quartile (Q3; 75^th^ percentile) and interquartile range (IQR). Item-specific number of respondents (n) varies because not all participants responded to every survey item.**
